# Heavy Metals in Agricultural Soils of the Lihe River Watershed, East China: Spatial Distribution, Ecological Risk, and Pollution Source

**DOI:** 10.3390/ijerph16122094

**Published:** 2019-06-13

**Authors:** Lian Chen, Genmei Wang, Shaohua Wu, Zhen Xia, Zhenang Cui, Chunhui Wang, Shenglu Zhou

**Affiliations:** 1 Guangzhou Marine Geological Survey, 477 Huanshi East Road, Guangzhou 510075, China; dg1527004@smail.nju.edu.cn (L.C.); cuizhenang@163.com (Z.C.); 2 School of Geography and Ocean Science, Nanjing University, 163 Xianlin Road, Nanjing 210023, China; zhousl@nju.edu.cn; 3 College of Forestry, Nanjing Forestry University, 159 Longpan Road, Nanjing 210037, China; 4 Institute of Land and Urban-Rural Development, Zhejiang University of Finance & Economics, Hangzhou 310018, China; shaohua@zufe.edu.cn; 5 College of Materials and Environmental Engineering, Hangzhou Dianzi University, Hangzhou 310018, China; wch@nju.edu.cn

**Keywords:** source apportionment, GIS mapping, enrichment factor, PMF, industrial and agricultural activity, parent material

## Abstract

Concentrations of cadmium, chromium, copper, nickel, lead, and zinc in agricultural soils at 32 sites in the Lihe River Watershed of the Taihu region, East China, and their potential ecological risks and possible sources were investigated. Enrichment factor analysis demonstrated enrichment in the order Cd > Pb > Zn > Cu > Ni > Cr. The potential ecological risk index and risk assessment code analyses indicated that, of the metals studied, Cd posed the most significant ecological risk in the study area. Statistical analyses, GIS mapping, and enrichment factor analysis suggested that Cd, Pb, Cu, and Zn were derived mainly from anthropogenic sources, including agricultural, industrial, and vehicular emissions, while Cr and Ni were mainly from natural sources. Positive matrix factorization revealed that Cd, Cr, Cu, Ni, Pb, and Zn were sourced from industrial and vehicular emissions (73.7%, 21.3%, 71.4%, 20.3%, 75.0%, and 62.2%, respectively), the agricultural sector (26.3%, 36.3%, 6.8%, 38.9%, 15.7%, and 6.9%, respectively), and parent materials (0%, 42.4%, 21.8%, 40.8%, 9.2%, and 30.9%, respectively). It was recommended that strategies be implemented to reduce industrial point-source pollution.

## 1. Introduction

Rapid urbanization and industrialization over recent decades have resulted in heavy metal contamination of agricultural soils, and heavy metal pollution of agricultural soil has become a worldwide environmental concern [1,2,3,4,5]. Heavy metal accumulation in soils can significantly affect human health through their environmental persistence, bioaccumulation, and transfer through food chains; consequently, control of pollution sources is required to minimize their impact [6]. The effective assessment of ecological risks and source apportionment is a crucial step in this regard.

Numerous analytical techniques have been applied in assessing ecological risks posed by heavy metals in agricultural soils, based on their concentration, distribution, and speciation [7,8,9]. Wang et al. [9] used the potential ecological risk index (PERI) to quantify degree of metal pollution and assess the ecological risk of heavy metals in soils. Although the PERI considers the toxicity and total concentrations of heavy metals, a degree of subjective assessment is involved and chemical speciation is ignored [8,10,11], even though toxicity and mobility are strongly dependent on the latter [12,13]. The risk assessment code (RAC) was introduced because of the advantage of comprehensive analysis of heavy metals and other components in soil, providing a better indication of relationships between bioavailability and mobility, as well as environmental risk [8,14]. Until now, there has been no systematic or integrated research focusing on ecological risk assessment of heavy metal contamination in the Lihe River Watershed in East China. In addition, the relevance of acid-soluble fractions of heavy metals, and source apportionment in this region, have not yet been investigated.

It is generally considered that heavy metals originate from two primary sources: natural (e.g., weathering of parental material) and anthropogenic (e.g., metalliferous industries, mining, vehicle exhausts, and agronomic practices) [1,2,15,16]. Existing methods of heavy metal source apportionment include descriptive statistical methods (coefficient of variation (CV) and correlation analysis), geographic information system (GIS) mapping, enrichment factor (EF) analysis, and positive matrix factorization (PMF). The CV, correlation, and EF analysis approaches determine roughly which heavy metals may be homologous, and which may be from anthropogenic or natural sources [17,18,19]. GIS mapping can estimate and quantify the spatial distributions of heavy metals [2,20,21,22,23], and provide additional information to distinguish point from non-point sources and identify potential pollutant sources [2,24,25]. However, these methods provide approximations only, and cannot definitively quantify the relative source contributions. PMF is therefore more widely used, because it estimates source component spectra based on a weighted-least-squares method and decomposes sample data into two matrices, namely the ‘factor-contribution’ and ‘factor-component’ matrices or spectra [26]. In previous studies, CV and correlation analysis, GIS mapping, EF analysis, and PMF have commonly been used alone, whereas the present study represents an attempt to integrate and apply all methods to a single site, thereby enabling cross-verification and the generation of more accurate and reliable results.

Unlike other studies, a research story in our study from pollution distribution to pollution assessment to sources apportionment was clearly and integrally described at sites in the Lihe River Watershed of the Taihu region, East China. Additionally, from the specific implementation details, a novel systematic risk assessment and a combined method of source apportionment for heavy metals were conducted. The aims were to (1) investigate the concentrations and spatial distribution patterns of Cd, Cr, Cu, Ni, Pb, and Zn in agricultural soils of the Lihe River Watershed; (2) assess the ecological risks posed by these metals based on combined PERI and RAC methods; and (3) identify the natural and anthropogenic sources of the metals and also calculate their contribution rate by combining CV and correlations analysis, GIS mapping, EF analysis, and PMF. The results provide insights into the management of heavy metal pollution in the Lihe River Watershed, and this study serves as a reference for other regions both in China and worldwide.

## 2. Materials and Methods

### 2.1. Study Area

The study area, in the Lihe River Watershed, is located to the west of Taihu Lake, Yixing City, Jiangsu Province, China. It includes two towns, Hufu and Dingshu, and is bounded by the coordinates 31°09′00″–31°20′31″ N and 119°42′00″–119°56′20″ E. Lihe River watershed has a surface area of ~260 km^2^, and agricultural land makes up 41% of the area [27]. There is a high density of industrial activity in the area, including a ceramics factory, a refractory materials plant, and a chemical plant, among others.

This study area was chosen because a previous study involving potential ecological risk assessment of heavy metal pollution in estuaries of the 24 main rivers flowing in and out Taihu Lake revealed that the Lihe River estuary was the most seriously contaminated [14]. However, there has been scant research into risk assessment and the source apportionment of heavy metals in this high-risk area.

### 2.2. Sample Collection and Preparation

**Figure 1:**
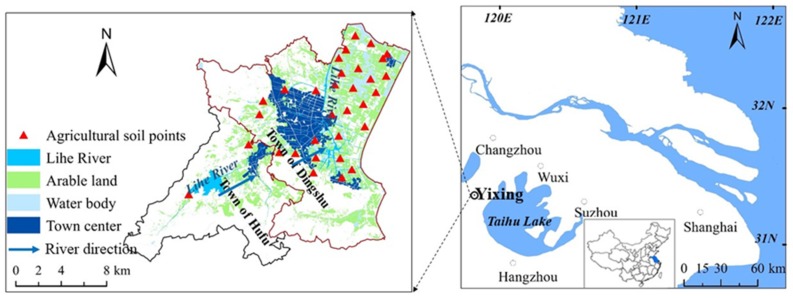
Location of the study area and distribution of sampling points.

Soils in crop fields were sampled at 0–10 cm depth from 32 randomly selected sites throughout the study area in May 2016 (Figure 1). The crop grown in the soil was wheat. Each sample of 0.5–1.0 kg was divided into 5–9 subsamples. The samples were air-dried at room temperature, ground, and passed through a 2 mm nylon sieve to remove stones and plant roots. The fine soil powders were then stored in polythene zip bags [28].

### 2.3. Chemical Analysis

#### 2.3.1. Soil Properties

Measurements of the particle size distribution were conducted using a MALVERN 2000 Laser particle size analyzer. The samples first received ultrasonic treatment in a 20% (NaPO_3_)_6_ solution, and then 3 cycles were measured with the analyzer to obtain 1/4 interval distribution of granularity data [3,4,29,30]; pH values of the soils were determined using a 10 g sample mixed with 25 mL of distilled water, which was stirred and left at room temperature (25 °C) for 0.5 h and then determined by a pH electrode [31]; loss-on-ignition method was used to analyze the soil organic matter (OM). Soil samples with constant weight were burned at 550 °C in a muffle in this process [32]; cation exchange capacity (CEC) was measured by the extraction method, using ammonium acetate (NH_4_OAc) and potassium chloride (KCl) [32]. A conductivity meter (Mettler Toledo) was used to measure the soil electrical conductivity (EC). Firstly, a suspension of 1:5 soil–water was configured and centrifuged for 30 min, and the clear supernatant extract was then used to test conductivity [33].

#### 2.3.2. Total Heavy Metal Concentrations

Total heavy metal concentrations were determined by HCl/HNO_3_/HF/HClO_4_ extraction. Approximately 100 mg of each sample was digested with 3 mL 37% HCl, 1 mL 65% HNO_3_, 6 mL 65% HF, and 0.5 mL 65% HClO_4_, using a two-stage digestion program involving heating for 10 min to 200 °C, and 15 min at 200 °C [34]. After cooling, the digestion solutions were evaporated to near dryness and the residue was dissolved in 1.0 mL 65% HNO_3_, after which deionized water (20 mL) was added. These solutions were stored in 25 mL high-density polyethylene vials at 4 °C until instrumental analysis [34]. Concentrations of Cd, Zn, Pb, Cu, Cr, and Ni in each solution were determined by inductively coupled plasma–mass spectrometry (ICP–MS; Perkin Elmer SCIEX, Elan 9000, Perkin Elmer Inc., America). Instrumental conditions are listed in Table S1 of the Supplementary Materials.

#### 2.3.3. Acid-Soluble Heavy Metal Fractions

Acid-soluble heavy metal fractions were determined by 0.1 mol L^–1^ HCl extraction [34]. Approximately 5 g of soil was placed in a pre-weighted 15 mL plastic centrifuge tube and the exact mass was recorded. HCl (5 mL, 0.1 mol L^–1^) was added and the tube was shaken for 4 h on an oscillating table. After shaking, the mixture was allowed to stand for 24 h before concentrations of Cd, Zn, Pb, Cu, Cr, and Ni were determined by ICP–MS.

#### 2.3.4. Quality Assurance and Control

Quality assurance (QA) and quality control (QC) involved analysis of replicate samples, method blanks, and standard reference materials, with duplicate analyses. Three replicate samples were analyzed, with relative standard deviations (RSD) for metal concentrations being no greater than 5% in all cases. All analyses were duplicated, and results are expressed as mean concentrations. Standard reference material GBW07405 (National Research Center for Standards in China) was used to verify analytical accuracy. All reagents were analytical grade or better. The lab glassware (bottles, tubes, etc.) was pre-cleaned by soaking in 10% HNO_3_ for at least two days, followed by soaking in and rinsing with de-ionized water prior to use.

### 2.4. Assessment of Soil Contamination

#### 2.4.1. Potential Ecological Risk Index

The PERI, introduced by Hakanson (1980), is used to assess the potential ecological risk of heavy metals in soil. This method not only assesses the pollution status of the soil, but also combines ecological and environment effects with toxicology, providing a better indication of potential risks denoted by index values. The potential ecological risk factor of a given metal (*E_i_*) is defined as:

(1) $$E_{i}=T_{i}\times (C_{i}/C_{0})$$

Equation (2) was used to calculate the total risk index (*RI*) of a sampling site, as follows:

(2) $$RI=\sum_{i=1}^{n}E_{i}$$

where *C_i_* is the concentration of metal *i* in soil, *C*_0_ is the background concentration, *T_i_* is the biological toxicity factor of an individual element (*T_i_* values are as follows: Cu = Pb = Ni = 5, Zn = 1, Cr = 2, and Cd = 30 [8,35,36], *E_i_* is the potential ecological risk factor of metal *i*, and *RI* is the total potential ecological risk index of metals *i–n*. The PERI of heavy metals was categorized according to five levels, as shown in Table 1.

#### 2.4.2. Risk Assessment Code

Assessment of soil heavy metal pollution was mainly based on the total concentration currently, and the speciation of heavy metals was often neglected. In fact, the acid-soluble fraction was the real reason for the risk. Therefore, methods of ecological risk assessment based on acid-soluble heavy metals, such as risk assessment code, have provided a new idea for ecological risk assessment of heavy metal pollution. Perin et al. put forward this method in 1985 [37]. The RAC was used to assess the risk and mobility of non-stable fractions of the metals [8], expressed as the percentage acid-soluble fraction (Table 2).

### 2.5. Source Identification and Apportionment

#### 2.5.1. Source Identification

##### GIS Mapping

GIS is a spatial information analysis technology that has developed rapidly in recent years. It has a powerful spatial analysis function, and is widely used in source identification. The greatest advantage of this technology is that it can convert huge amounts of data information into a more intuitive spatial distribution map in the process of source identification, and utilize the spatial distribution maps to qualitatively identify the pollution sources. In general, GIS mapping can distinguish point source pollution from non-point source pollution [38]. In spatial analysis using ArcMap 10.0 software, the Kriging method was used for spatial interpolation.

##### Coefficient of Variation (CV)

When comparing the degrees of dispersion of two groups of data, it is not appropriate to use the standard deviation (SD) if the dimensions of the two groups are different. The CV overcomes this problem because it involves the ratio of the standard deviation of raw data to the mean value, as follows:

(3) $$\mathrm{CV}=\frac{\mathrm{SD}}{\mathrm{Mean}}$$

Coefficient of variation can be divided into three grades: low variation (CV < 15%), medium variation (15% < CV < 36%), and high variation (CV > 36%) [39].

Coefficient of variation of heavy metal concentration can reflect the interference degree of human activities to environmental media. Generally speaking, environmental media that is strongly affected by human activities may produce a higher coefficient of variation of heavy metal concentration [39]. There are similarities between source identification by calculating coefficient of variation and by using GIS mapping. A high coefficient of variation and an uneven spatial distribution of heavy metals concentration can indicate a high proportion of human sources.

##### Correlation Analysis of Heavy Metals

Correlation analysis is a relatively simple and convenient method for source identification of heavy metal pollution, and it is one of the most widely used methods currently. Through the correlation analysis of heavy metals, some effective information about the pollution sources has been obtained. It can provide some important information for the further analysis of pollution sources, and also can verify the results of source apportionment obtained by other methods. In general, when the concentrations of two heavy metals are significantly positively correlated, the two heavy metals may be homologous, and when they are negatively correlated, they may have different sources [40]. In this study, Pearson correlation analysis was used in SPSS software. Remarkably, most multivariate statistical methods require variables to conform to the normal distribution. Thus, the normality of the distribution of each variable was checked by analyzing kurtosis and skewness statistical tests before Pearson correlation analysis [41,42].

##### Enrichment Factors (EF)

Enrichment factor (EF) is a useful tool for determining the degree of anthropogenic heavy metal pollution [11,23]. It was used to describe metal enrichment in agricultural soil, because it is necessary to reduce the effect of grain size on contamination through the normalization of concentration data with respect to a conservative element [9,43]. Iron is inert in migration processes and is derived mainly from natural lithogenic sources, so it is generally recognized as being an appropriate normalization element [44]. The EF values for elements in soil samples were thus calculated as follows [44]:

(4) $$EF=\frac{(C_{i}/C_{Fe})sample}{(C_{i}/C_{Fe})crust}$$

where *C_i_* is the element concentration in the sample or continental crust, and *C_Fe_* is the concentration of the reference element (Fe). Background values for soils in Jiangsu Province were taken as continental crust values: Fe 3.02%, Cd 0.126 mg kg^−1^, Cr 77.8 mg kg^–1^, Cu 22.3 mg kg^–1^, Ni 26.7 mg kg^–1^, Pb 26.2 mg kg^–1^, and Zn 62.6 mg kg^–1^ [45]. An EF value of 0.5–1.5 indicates that a metal is derived mainly from crustal materials or natural weathering processes, while EF > 1.5 indicates that a significant portion originated from non-crustal or anthropogenic processes [46].

#### 2.5.2. Source Apportionment by Positive Matrix Factorization (PMF)

The above methods only qualitatively identify the major sources of heavy metals in soil. However, it is impossible to quantitatively calculate the contribution rate of each type of pollution source using them. PMF analysis can achieve the goal of source apportionment.

PMF analysis employed the US EPA PMF 5.0 model for soil pollutant source apportionment, as developed by Paatero and Tapper [47]. The PMF is a typical receptor model and can be used without source composition as an input. The notable features of this approach are non-negativity constraints in obtaining physically realistic meanings, and the use of uncertainty to weight each data point individually [48]. Furthermore, PMF can supplement values that are missing or below detection limits to ensure the reliability of each data point [26].

The PMF model defines an n × m original data matrix *X*, where n represents the number of samples and m the number of chemical species, which can be factorized into two sub-matrices, namely *G* (n × p) and *F* (p × m), with an unexplained part *E* (n × m) [26,49], as follows:

*X* = *G* + *F* (5)

(6) $$x_{ij}=\sum_{k=1}^{p}g_{ik}f_{kj}+e_{ij}$$

where *x_ij_* is the concentration of the *j*th chemical species measured in the *i*th sample; *g_ik_* is the contribution of source *k* to the ith sample; *f_kj_* is the concentration of the *j*th chemical species in source *k*; and *e_ij_* is the residual for each sample and species. The residual error matrix, *e_ij_*, is obtained by minimizing the function *Q*, where

(7) $$Q={\sum_{i=1}^{n}\sum_{j=1}^{m}\left(\frac{e_{ij}}{u_{ij}}\right)}^{2}$$

and *u_ij_* is the uncertainty of the *j*th chemical species for sample *i*. The model can be optimized by including the uncertainty, *u*, for each sample, calculated using the measurement uncertainty (MU) and detection limit (MDL). When the metal concentration is ≤MDL, *u* is calculated as

(8) $$u=\frac{5}{6}\times \mathrm{MDL}$$

and when the concentration is >MDL, we have

(9) $$u=\sqrt{{\left(\mathrm{MU}\times \mathrm{concentration}\right)}^{2}+{\left(\mathrm{MDL}\right)}^{2}}$$

## 3. Results and Discussion

### 3.1. Concentrations of Heavy Metals in Soils

#### 3.1.1. Descriptive Statistics of Soil Properties

Descriptive statistics of soil properties are shown in Table 3. The pH of the soil ranged from 4.89 to 7.45, with an average of 5.81. The proportion of samples in which pH was less than 7 among the 32 soils was 93.75%. Soil cation exchange capacity (CEC) is an important factor reflecting soil buffer capacity, and also an important basis for evaluating soil nutrient holding capacity, improving soil equality, and rational fertilization. The average soil cation exchange capacity in the study area was 18.41 cmol·kg^−1^, which belongs to a medium-upper level. Soil electrical conductivity (EC) is an index for determining water-soluble salts in soil, and it is a factor to determine whether salt ions in soil restrict crop growth or not. Different plants have different optimum intervals for soil conductivity. The average conductivity of soil in the study area was 1.59 ms/cm. Soil organic matter content (OM) is an index reflecting soil fertility. According to soil fertility classification recommended by the second national soil census, if the organic matter of a soil is less than 1%, it is organic matter deficient soil. There was no sample in which the organic matter was less than 1%, which indicated that the soil fertility in the study area was relatively good. Soil particle size was related to soil adsorption capacity of heavy metals. Generally, the smaller the particle size of soil is, the stronger the adsorption capacity and the higher the heavy metal concentration would be. The average soil particle size in the study area was 24.37Φ.

#### 3.1.2. Descriptive Statistics of Total Heavy Metal Concentrations

Basic statistics related to the total heavy metal concentrations in soils from the Lihe River Watershed and other selected areas are summarized in Table 4, together with background values of soil in Jiangsu Province and standard values (Soil Environmental Quality Standard Two, SEQ-II, GB 15618-1995, grade II for agricultural land). SEQ-II values are threshold values for the protection of human health and agricultural production in China. A wide range of total heavy metal concentrations was observed in soils across the study area (Table 4), and mean Cd, Cu, Pb, and Zn concentrations were all higher than their respective background values (the Cd concentration was four times the background value). Mean Cr and Ni concentrations were lower than their background values. The Cd, Cr, Cu, Ni, Pb, and Zn concentrations exceeded background values in 100%, 3%, 31%, 25%, 94%, and 94% of the 32 soil samples, respectively. Comparison of mean metal concentrations with SEQ-II values indicates that Cd pollution is the most serious, with a concentration almost twice the SEQ-II value, indicating that anthropogenic sources have a direct effect on soil Cd concentrations [50]. The CV values of metal concentrations followed the order Cd (0.72) > Pb (0.52) > Cu (0.42) > Zn (0.39) > Ni (0.20) > Cr (0.17).

In comparisons with other regions (Table 4) the mean concentration of Cd in the Lihe River Watershed (0.57 mg kg^–1^) was higher than that in the Yangtze and Pearl river systems (0.19 and 0.32 mg kg^–1^, respectively [9,51], while concentrations of the other metals were both higher and lower. This indicates that the problem of high Cd levels in soil of the Lihe River Watershed requires attention.

**Figure 2:**
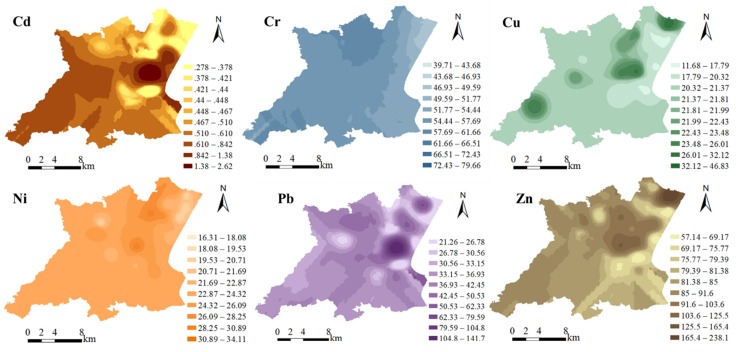
Spatial distributions of Cd, Cr, Cu, Ni, Pb, and Zn concentrations (mg/kg) in the study area. The spatial distributions of elements were determined with the kriging interpolation method.

Spatial distributions of the metals are shown in Figure 2, revealing that the distributions of Cd, Pb, Cu, and Zn are largely similar, being higher in central-eastern and southern parts of the study area. Cr and Ni are distributed relatively uniformly over the whole study area, although they occur at slightly higher levels in the west and lower levels in the east.

#### 3.1.3. Acid-Soluble Heavy Metal Concentration

The ratio of acid-soluble concentration to total concentration of six heavy metals can reflect the mobility of heavy metals in soil. Descriptive statistics of acid-soluble heavy metal concentrations are shown in Table 5. The order of acid-soluble concentrations of the six heavy metals was Pb > Cu > Cd > Zn > Ni > Cr, which indicated that the mobility of Pb and Cu was relatively high, while that of Ni and Cr was low. The result was consistent with the study by Dario et al. [51]. This suggested that for Cr and Ni, the input ratio of anthropogenic sources was relatively low, while it was high for Pb and Cu. It is noteworthy that the mobile heavy metals in soil are easily absorbed by plants, and eventually enter the human body through the food chain, which poses a great threat to the human body. Therefore, Pb, Cu, Cd, and Zn in the soil of the study area are the key heavy metals that posed a threat to the human body and need to be paid attention, while the threat of Cr and Ni to the human body is relatively small.

#### 3.1.4. Correlation between Heavy Metal Concentration and Soil Properties

The correlation between heavy mental concentration and soil properties is shown in Table 6. It was suggested that the total concentration of Cr was significantly correlated with CEC and pH. The available concentrations of Cr and Ni were positively correlated with CEC at 0.01 level. However, for other heavy metals, there was no correlation between the concentration and soil properties. Thus, Cr and Ni were more affected by soil properties. Whether this result indicates that Cr and Ni derived mainly from parent material is a question worthy of further verification.

### 3.2. Risk Assessment of Heavy Metals in Soil

#### 3.2.1. Potential Ecological Risk Indices

Potential ecological risks of the metals were calculated using Equations (1) and (2), with results presented in Table 7. Mean potential ecological risks are ranked as Cd > Pb > Cu > Ni > Zn > Cr. The *E_i_* values (Equation (1)) for Cr, Cu, Ni, Pb, and Zn are all <40, indicating low potential ecological risk, whereas Cd (*E_i_* = 131.9) poses a considerable risk. The mean *RI* value (Equation (2)) for the study area indicates a moderate risk, to which the *E_i_* value for Cd contributes 86.8% of the potential ecological risk.

#### 3.2.2. Risk Assessment Code

The acid-soluble fraction indicates the degree of mobility of heavy metals in soil, further reflecting ecological risk, and follows the order Pb > Cu > Cd > Zn > Ni > Cr (Table 8). According to the RAC classification (Table 2), Pb, Cu, and Cd exhibit the highest mobility and therefore the greatest potential bio-available risk, with 43–68% solubility. The lower acid solubility of Zn and Ni indicate medium risk, with low risk for Cr. Overall, the environmental risk of the bio-available fraction, based on RAC values, decreases in the order Pb > Cu > Cd > Zn > Ni > Cr, indicating that Pb, Cu, and Cd pose the greatest ecological risk in the Lihe River Watershed.

### 3.3. Identification and Apportionment of Pollution Sources

#### 3.3.1. Identification of Pollution Sources

##### GIS Mapping of Heavy Metals

GIS mapping can assist in distinguishing point from non-point sources, and in identifying potential pollutant sources [2,20,21,24]. The higher Cd, Pb, Cu, and Zn concentrations tend to be clustered in circular patterns around specific points (Figure 2), indicating point-source pollution. In contrast, Cr and Ni concentrations are relatively uniform, with no obvious low or high values in the study area, indicating geological background or non-point sources.

##### Coefficient of Variation of Metal Concentrations

Generally, the greater the CV, the higher the contribution of anthropogenic sources to metals in soils [40]. Therefore, the calculated CV values (Table 4) indicate that Cd, Pb, Cu, and Zn (CV = 0.39–0.72) are derived mainly from anthropogenic sources, while Cr and Ni (CV = 0.17–0.20) are mainly crustal in origin.

##### Correlations between Metals

Inter-elemental relationships can provide information regarding pollution sources [52], as the heavy metals of most interest commonly have a shared origin [53]. Before correlation analysis, the normality of the distribution of heavy metal concentration was checked [42]. Through analysis in SPSS, our statistical data were conformed to normal distribution. Pearson correlation coefficients for metal concentrations (Table 9) indicate highly positive correlations (*p* ≤ 0.01) between Cd and Pb (r = 0.84), Cr and Ni (r = 0.68), Cu and Zn (r = 0.76), and Cu and Pb (r = 0.62), indicating that these pairs of metals may have the same origin.

##### Enrichment Factors

The statistics of the EFs for all the metals analyzed were evaluated to further clarify the potential sources of metals in soils in the study area. The EF values for Cd are highest, with a mean of 15.56, and values for Pb and Zn are moderate, with means of 2.63 and 2.58, respectively. Mean EF values (and CV values) follow the order Cd > Pb > Zn > Cu > Ni > Cr. Cd, Pb, Zn, and Cu are thus considered to be enriched (EF = 1.75–15.56), while Cr and Ni show relatively low enrichment (EF = 1.19–1.53). Considered together, the mean EF and CV values (Table 10) indicate that Cd, Pb, Cu, and Zn have anthropogenic origins, whereas Cr and Ni are derived from the crust.

#### 3.3.2. Source Apportionment by PMF

The PMF model was run using between three and six factors, and each run was initialized with different starting points. A random-seed mode with 20 random starting points was selected and 3–6 factors were examined. PMF analysis identified four appropriate factors among the soils (Table 11). Tentative identification of the factor profiles was based on elemental markers in the different sources.

Source composition profiles based on a four-factor solution are provided in Table 11. Factor 1 is dominated by Cd and accounts for 52.11% of the Cd source. The EF value for Cd is the highest of the six metals, averaging 15.56, which indicates Cd is significantly enriched in the soil. In addition, the results of the PERI and RAC assessments indicate that Cd poses a high ecological risk. The widely distributed ceramics industries in the study area use large amounts of Cadmium Yellow pigment, which may be an important source of the Cd pollution. It is clear, therefore, that the first factor represents a source related to the ceramics industry.

The second factor is dominated by Cu, Pb, and Zn. Concentrations of these metals are higher than background values, with mean EF values of 1.75, 2.63, and 2.58 (Table 8), respectively, which indicate moderate enrichment in soil. The spatial distributions of Cu, Pb, and Zn in the study area are uneven (Figure 2). Previous studies have indicated that industrial and vehicular sources are mainly responsible for Cu, Pb, and Zn inputs to soils and the environment [54], with Cu being mainly from machinery manufacturing plants, Zn (a hardness additive) from tire dust, and Pb from coal combustion and automobile exhaust emissions [53,55]. Since the phasing out of leaded petrol, the input of Pb to the environment has reduced, but vehicle brakes and tires still release Pb, meaning that vehicular emissions remain an important source of pollution [54]. The development of coal-fired power stations and heavy vehicle traffic have accompany industrialization in the study area, so the second factor can be attributed to industrial activity and vehicular emissions.

In the third factor, all of the elements have some loading, except for Ca. The large-scale use of agricultural chemicals (e.g., manure, fertilizers, and pesticides) may cause the enrichment of many elements in soil, including Cd, Cr, Cu, Ni, Pb, Zn, Co, Mn, K, and Mg [26,53,55,56]. Some of these elements, such as Cu, Ni, Pb, Zn, Co, Mn, K, and Mg, are important nutrients in crop production, and are provided primarily by commercial fertilizers to improve the soil fertility. Some, such as Cd, Cr, and Pb, are not essential for plant growth and are not intentionally introduced. PMF analysis thus indicates that the third factor represents sources based on agronomic practices.

For the fourth factor, Ca, Co, Cr, Fe, K, Mg, Mn, and Ni received higher weightings than other metals. These elements, in particular Ca, K, Mg, and Mn, are associated with crustal material. Minerals in the topsoil of the study area comprise mainly quartz, potassium feldspar, and anorthoclase, which contain Al2O3, CaO, MgO, SiO2, and K2O [26], and these oxides are released during weathering. Concentrations of Cr and Ni are slightly lower than background values, and their uniform distribution in the study area and low CVs indicate that they have low spatial variability. The mean EF values of Cr and Ni are ≤1.5, indicating minimal enrichment. In addition, PERI and RAC assessments indicate that Cr and Ni pose low or zero potential ecological risk. This group of elements is thus likely to originate from natural sources, such as mineral weathering and atmospheric precipitation. This is consistent with the findings of previous studies [7,8,26,53,56].

The present results concerning heavy metal source identification are largely consistent with those of previous studies. Xue et al. [26] combined PMF with a geo-statistical approach in Changxing County, Zhejiang Province, and found that Cd, Cu, Pb, and Zn were mainly from anthropogenic sources, while Cr and Ni were mainly from natural sources. Dai et al. [56] used multivariate analysis and geostatistics to identify the sources of heavy metals in Laiwu City, Shandong Province, Eastern China, and found that Cd, Cu, Pb, and Zn, were dominated by industrial, agricultural, and vehicular sources, while Cr and Ni came from natural sources and were controlled by parent materials. Peris et al. [57] performed a multivariate statistical analysis of agricultural soils in the European Mediterranean region, and found that Ni was mainly associated with parental rocks, while Cd, Cu, Pb, and Zn were connected with anthropogenic activity. Using a combination of GIS mapping and multivariate statistical analysis, Cai et al. [53] demonstrated that Ni and Cr originate from natural sources, whereas Cd and Pb are mainly derived from anthropogenic activity. Li et al. [7] used correlation and multivariate statistical analysis to show that Cu, Cd, Pb, and Zn concentrations in soils were mainly anthropogenic in origin, whereas Cr and Ni concentrations were mainly influenced by natural factors, such as parent materials.

**Figure 3:**
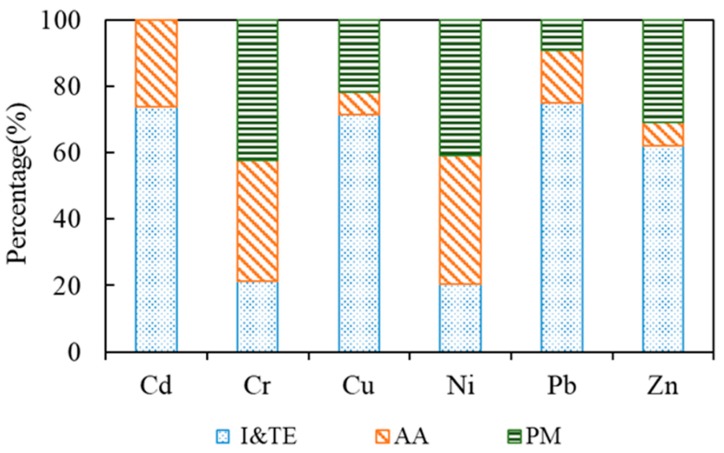
Relative source contributions to the heavy metals analyzed in the present study. I&TE, AA, and PM represent industrial and vehicular emissions, agricultural activity, and parent materials, respectively.

Source contribution rates were also determined by PMF (Figure 3). Factor 1 (representing industrial activity related to the ceramics industry) and factor 2 (industrial and vehicular emissions) were combined. The total contributions of industrial and vehicular emissions to Cu, Pb, and Zn levels in the agricultural soils are 71.4%, 75.0%, and 62.2%, respectively, while agricultural sources contribute 6.8%, 15.7%, and 6.9%, respectively, and parent materials contribute 21.8%, 9.2%, and 30.9%, respectively. For Cr and Ni, the contribution rates of parent material are highest, followed by agricultural activity and industrial and vehicular emissions. For Cd, industrial and vehicular emissions, and agricultural sources account for 73.7% and 26.3%, respectively. There is no natural source of Cd in the study area, and because Cd pollution is overwhelmingly identified as the most significant ecological risk factor in the study area, it should have the highest priority in controls imposed on heavy metal pollution by industrial sources.

## 4. Conclusions

This study analyzed the spatial distribution, ecological risks, and sources of the heavy metals Cd, Cr, Cu, Ni, Pb, and Zn in agricultural soils in the Lihe River Watershed. Average concentrations of Cd, Cu, Pb, and Zn were all higher than background values, with the mean Cd concentration being four times higher and almost twice the SEQ-II value. Average Cr and Ni concentrations were lower than background values. Cd, Cu, Pb, and Zn exhibit similar spatial distributions, with concentrations clearly higher in the central-eastern and southern parts of the study area. In contrast, Cr and Ni have relatively uniform distributions across whole study area, although slightly higher in the west and slightly lower in the east. Based on the results of PERI and RAC assessments, it was concluded that Cd poses a considerable ecological risk, and it was identified as the most significant factor affecting the ecological environment of the study area.

Source identification based on the combined methods of GIS mapping and CV, correlation, and EF analyses indicates that Cd, Pb, Cu, and Zn are derived mainly from anthropogenic sources, including agricultural, industrial, and vehicular emissions, while Cr and Ni originate mainly from natural sources. Source apportionment by PMF analysis indicates that for Cd, Cr, Cu, Ni, Pb, and Zn in the agricultural soils, industrial and vehicular emissions contribute 73.7%, 21.3%, 71.4%, 20.3%, 75.0%, and 62.2%, respectively; agricultural sources contribute 26.3%, 36.3%, 6.8%, 38.9%, 15.7%, and 6.9%, respectively; and parent materials contribute 0%, 42.4%, 21.8%, 40.8%, 9.2%, and 30.9%, respectively. Important strategies should be implemented to reduce the use of chemical fertilizers and pesticides and, even more importantly, control industrial point-source pollution to reduce ecological risks associated with Cd pollution.

## Figures and Tables

**Table 1:** Classification of potential ecological risk index (PERI).

Assessment Criterion	PERI				
	Low	Moderate	Considerable	High	Very High
*E_i_*	<40	40–80	80–160	160–320	≥320
*RI*	<150	150–300	300–600	≥600	

**Table 2:** Classification of risk assessment code (RAC).

Risk	Acid Soluble Fraction (%)
No risk	<1
Low risk	1–10
Medium risk	11–30
High risk	31–50
Very high risk	51–75

**Table 3:** Soil properties including pH, cation exchange capacity (CEC), electrical conductivity (EC), organic matter (OM), and particle size.

Soil Properties	Range	Mean Value	Standard Deviation	CV
pH	4.85–7.45	5.81	0.69	0.12
CEC (cmol·kg^−1^)	11.76–22.99	18.41	2.60	0.14
EC (ms·cm^−1^)	0.67–3.05	1.59	0.65	0.41
OM (%)	1.84–5.35	3.58	0.94	0.26
Average particle size (Φ)	8.45–52.06	24.37	8.37	0.34

CV: Coefficient of Variation.

**Table 4:** Total heavy metal concentrations in soil from the Lihe River Watershed and other selected areas for comparison (unit mg kg^–1^).

Heavy Metals	The Lihe River Watershed						Background Values of Soil in Jiangsu Province	SEQ-II	The Yangtze River Estuary, China
	Range	Mean ± SD	Median	First Quartile	Third Quartile	CV			Mean
Cd	0.28–2.62	0.57 ± 0.41	0.45	0.40	0.59	0.72	0.13	0.3	0.19
Cr	39.71–79.66	54.60 ± 9.04	53.95	47.64	59.61	0.17	77.8	200	79.1
Cu	11.68–46.83	22.71 ± 9.44	21.17	16.09	22.96	0.42	22.3	100	24.7
Ni	16.31–34.11	24.35 ± 4.88	24.05	21.10	27.17	0.20	26.7	50	31.9
Pb	21.26–141.72	39.78 ± 20.61	36.75	29.10	43.13	0.52	26.2	300	23.8
Zn	57.14–238.06	93.29 ± 38.50	82.61	76.08	93.45	0.39	62.6	250	82.9
References	Present study						Chen et al. [4]	Chen et al. [4]	Wang et al. [9]

**Table 5:** The available concentration of six heavy metals in the soil.

Heavy Metals	Range	Mean Value ± Standard Deviation	Coefficient of Variation
Cd	0.11–1.54	0.25 ± 0.25	98.16
Cr	0.60–1.85	1.27 ± 0.26	20.01
Cu	7.45–34.00	14.85 ± 7.09	47.76
Ni	1.16–4.18	2.64 ± 0.77	29.29
Pb	12.51–91.84	26.84 ± 13.61	50.73
Zn	9.32–59.10	18.92 ± 11.44	60.46

**Table 6:** The correlation between heavy mental concentration and soil properties.

Heavy Metals	pH	CEC	EC	OM	Average Particle Size
Cd-s	0.222	0.123	0.287	0.132	0.033
Cr-s	0.444 *	0.449 **	0.316	0.266	−0.223
Cu-s	0.229	0.075	0.146	0.076	0.103
Ni-s	0.041	0.040	0.024	−0.098	−0.131
Pb-s	0.098	0.115	0.255	0.182	−0.038
Zn-s	−0.008	−0.117	−0.086	−0.226	−0.113
Cd-sa	0.199	0.081	0.216	0.122	0.072
Cr-sa	0.215	0.627 **	0.301	0.285	−0.334
Cu-sa	0.123	0.049	0.075	0.067	0.184
Ni-sa	−0.120	0.457 **	0.130	0.032	−0.154
Pb-sa	0.044	0.124	0.247	0.194	−0.062
Zn-sa	−0.062	−0.242	−0.111	−0.309	−0.104

Notes: “Cd-s” denotes the concentration of Cd in soil; “Cd-sa” denotes the available concentration of Cd in soil. It was same as “Cr, Cu, Ni, Pb, and Zn.” In addition, “**” indicates correlation was significant at the 0.01 level (two-tailed), while “*” indicates correlation was significant at the 0.05 level (two-tailed). CEC: cation exchange capacity; EC: electrical conductivity; OM: organic matter.

**Table 7:** Descriptive statistics of *E_i_* and *RI* for heavy metals in the study area.

Parameter	Heavy Metal	Mean	Max	Min	SD	CV	Risk Classification
*E_i_*	Cd	131.9	604.4	64.16	95.26	0.722	Considerable risk
	Cr	1.403	2.048	1.021	0.232	0.166	Low risk
	Cu	5.093	10.50	2.619	2.117	0.416	Low risk
	Ni	4.560	6.388	3.054	0.914	0.200	Low risk
	Pb	7.592	27.04	4.057	3.933	0.518	Low risk
	Zn	1.490	3.803	0.913	0.615	0.413	Low risk
*RI*	(Multi-metal)	152.0	649.5	300-600	99.79	0.656	Moderate risk

SD: Standard Deviation; CV: Coefficient of Variation.

**Table 8:** Acid-soluble fraction of heavy metals in soil and their risk classifications.

Heavy Metals	Acid Soluble Fraction (%)	Risk Classification
Cd	43.02 ± 10.35	High risk
Cr	2.36 ± 0.46	Low risk
Cu	65.19 ± 10.29	Very high risk
Ni	11.25 ± 4.05	Medium risk
Pb	67.72 ± 6.97	Very high risk
Zn	19.79 ± 4.72	Medium risk

**Table 9:** Pearson correlation coefficients for heavy metals (n = 32).

Heavy Metals	Cd	Cr	Cu	Ni	Pb	Zn
Cd	1	−0.012	0.450 ^a^	0.121	0.841 ^a^	0.079
Cr		1	0.053	0.677 ^a^	−0.194	−0.045
Cu			1	0.040	0.617 ^a^	0.763 ^a^
Ni				1	−0.013	−0.103
Pb					1	0.345
Zn						1

^a^ Correlation is significant at the 0.01 level (two-tailed).

**Table 10:** Descriptive statistics for the enrichment factors (EF) of heavy metals and their coefficient of variation (CV) values.

Heavy Metal	Mean ± SD	Range	CV
Cd	15.56 ± 11.68	7.92–74.03	0.75
Cr	1.19 ± 0.12	0.95–1.45	0.10
Cu	1.75 ± 0.80	0.88–3.88	0.45
Ni	1.53 ± 0.18	1.29–2.16	0.12
Pb	2.63 ± 1.46	1.22–9.63	0.56
Zn	2.58 ± 1.28	1.51–7.84	0.50

**Table 11:** Source contribution for different elements by positive matrix factorization (PMF).

Elements	Profile Contribution (mg/kg)				Percentage Contribution (%)			
	Factor 1	Factor 2	Factor 3	Factor 4	Factor 1	Factor 2	Factor 3	Factor 4
Ca	196.84	2.9 × 10^−15^	5.9 × 10^−6^	2220.70	8.14	1.2 × 10^−16^	2.4 × 10^−7^	91. 86
Cd	0.30	0.12	0.15	2.0 × 10^−13^	52.11	21.62	26.27	3.5 × 10^−11^
Co	0.92	4.71	7.24	8.17	4.38	22.37	34.43	38.82
Cr	0.21	11.23	19.52	22.82	0.40	20.88	36.30	42.43
Cu	1.51	14.03	1.48	4.75	6.92	64.44	6.81	21.82
Fe	254.28	3477.7	6417.3	7712.2	1.42	19.47	35.93	43.18
K	69.38	2557.1	3587.2	5308.9	0.60	22.19	31.13	46.07
Mg	5.3 × 10^−5^	491.51	982.34	1624.2	1.7 × 10^−6^	15.87	31.71	52.43
Mn	19.46	52.68	45.14	140.26	7.55	20.46	17.53	54.46
Ni	0.50	4.33	9.27	9.70	2.10	18.19	38.94	40.77
Pb	9.74	18.90	6.01	3.53	25.51	49.50	15.74	9.25
Zn	0	56.52	6.24	28.10	0	62.21	6.87	30.93

## Supplementary Materials

The following are available online at https://www.mdpi.com/1660-4601/16/12/2094/s1, Table S1: Operating parameters for ICP-MS (ELAN 9000, PerkinElmer SCIEX) for the determination of elemental concentrations, Figure S1: The distribution of heavy metal concentration in the study area.
