# Supplementary material for: Heavy Metals in Agricultural Soils of the Lihe River Watershed, East China: Spatial Distribution, Ecological Risk, and Pollution Source
Source: Int J Environ Res Public Health. 2019 Jun 13;16(12):2094. doi: 10.3390/ijerph16122094 (PMC6617031; doi:10.3390/ijerph16122094)
Supplement: Supplementary file 1 [file ijerph-16-02094-s001.pdf]

Supplementary Materials

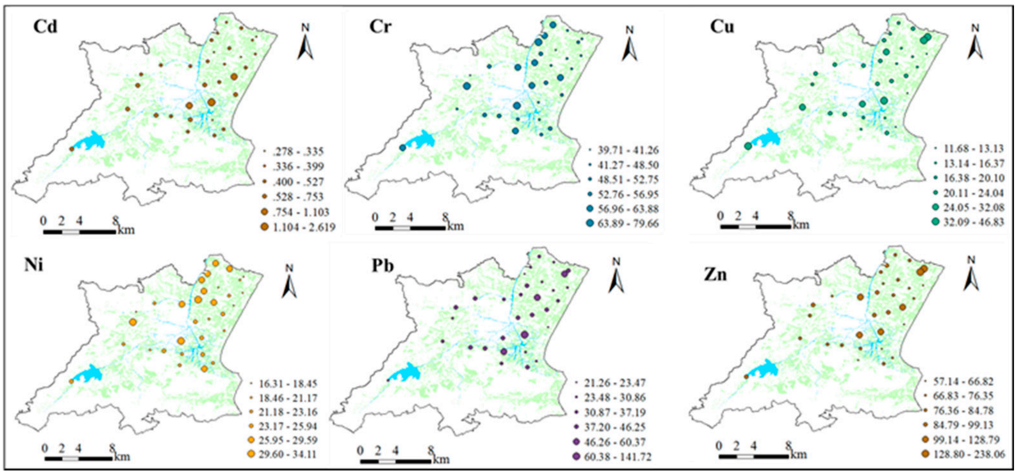

Figure S1. The distribution of heavy metal concentration in the study area.

Table S1. Operating parameters for ICP-MS (ELAN 9000, PerkinElmer SCIEX) for the determination of elemental concentrations.

Parameter	Value	Parameter	Value
Nebulizer gas flow (L min^{-1})	0.94	RF power (W)	1100
Analog stage voltage (V)	−1700	Lens voltage (V)	6
Pulse stage voltage (V)	900	Ac rod offset (V)	−6
Discriminator threshold (V)	70	Scan mode	Peak hopping
Speed of peristaltic pump (rpm)	26	Detector	Pulse
Sweeps/Reading	50	Replicates	2
Sampler/Skimmer cones	Nickel	Dwell Time (ms)	2.5
Spray chamber	Ryton® Double-pass Scott-type spray chamber		
Nebulizer	Gem-tip Cross-Flow pneumatic nebulizer		
